# A scoping review of mentor training programs in medicine between 1990 and 2017

**DOI:** 10.1080/10872981.2018.1555435

**Published:** 2018-12-31

**Authors:** Krish Sheri, Jue Ying Joan Too, Sing En Lydia Chuah, Ying Pin Toh, Stephen Mason, Lalit Kumar Radha Krishna

**Affiliations:** a Yong Loo Lin School of Medicine, National University of Singapore, Singapore; b Department of Family Medicine, National University of Singapore, Singapore; c University of Liverpool, Marie Curie Palliative Care Institute, Liverpool, UK; d Division of Palliative Medicine, National Cancer Centre Singapore, Singapore; e Centre of Biomedical Ethics, National University of Singapore, Singapore; f Duke- NUS Medical School, Singapore

**Keywords:** Mentor, mentor training, medical education, mentoring framework, undergraduate mentoring, postgraduate mentoring

## Abstract

Effective mentoring enhances the personal and professional development of mentees and mentors, boosts the reputation of host organizations and improves patient outcomes. Much of this success hinges upon the mentor’s ability to nurture personalized mentoring relationships and mentoring environments, provide effective feedback and render timely, responsive, appropriate, and personalized support. However, mentors are often untrained raising concerns about the quality and oversight of mentoring support.

To promote effective and consistent use of mentor training in medical education, this scoping review asks what mentor training programs are available in undergraduate and postgraduate medicine and how they may inform the creation of an evidenced-based framework for mentor training.

Six reviewers adopted Arksey and O’Malley’s approach to scoping reviews to study prevailing mentor-training programs and guidelines in postgraduate education programs and in medical schools. The focus was on novice mentoring approaches. Six reviewers carried out independent searches with similar inclusion/exclusion criteria using PubMed, ERIC, EMBASE, SCOPUS, Google Scholar, and grey literature databases. Included were theses and book chapters published in English or had English translations published between 1 January 1990 and 31 December 2017. Braun and Clarke’s approach to thematic analysis was adopted to circumnavigate mentoring’s and mentor training’s evolving, context-specific, goal-sensitive, learner-, tutor- and relationally dependent nature that prevents simple comparisons of mentor training across different settings and mentee and mentor populations.

In total, 3585 abstracts were retrieved, 232 full-text articles were reviewed, 68 articles were included and four themes were identified including the structure, content, outcomes and evaluation of mentor training program.

The themes identified provide the basis for an evidence-based, practice-guided framework for a longitudinal mentor training program in medicine and identifies the essential topics to be covered in mentor training programs.

## Introduction

Novice mentoring dominates the mentoring landscape in medical education [1–6] and has been found to enhance innovation and career progression [7–15], encourage research involvement amongst women and under-represented ethnic minorities, boost grant success [16], and publications [10,17–23] and navigating the complex landscape of academic life [2,24–27]. Clinicians who have been mentored are also more motivated, resilient, have well-developed professional identities and feel better supported in their jobs than colleagues without mentors [28–31]. Mentoring also has a role in portfolio-learning [32,33] and competency-based curricula [34–38].

**Figure 1.**
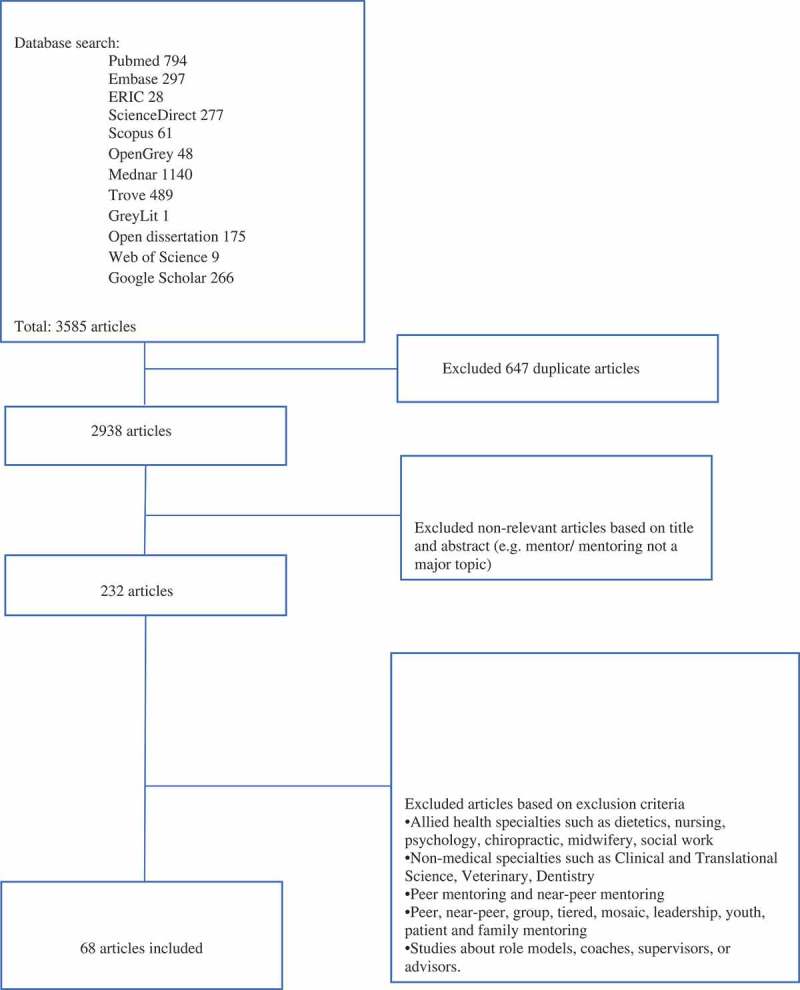
PRISMA flowchart.

However, despite its success and expanded use in medical education, clinicians are poorly trained in the art of mentoring [24,34]. Feldman et al. (2009) reported that less than 15% of mentors received formal mentor training and Shea et al. (2011) noted that less than one-third of institutions have inclusion criteria for mentor recruitment [17,22]. Unsurprisingly mentors often fail to maximise mentoring opportunities [24,34]. Acknowledging this gap, Geraci and Thigpen (2017) emphasised the need for mentor training where mentors ‘*engage in self-reflection and assessment to determine if in fact they have the attitudes, personal qualities, knowledge and skills and can regularly demonstrate the behaviours that are needed to maximize protégé success*’ [39]. Mentor training [11,40,41] enhances the effectiveness of the mentoring process [19,23,39,42,43], increases mentoring competency [3,4,11,44–48], improves mentor satisfaction [49], and boosts staff retention [3,4,46,50].

However, neither a clear syllabus nor an effective approach to mentor training exists [1,3–6,51]. These gaps are attributed to differences in the understanding, practice, goals and context of mentoring in medical education, unique curricula, diverse mentee and mentor populations, and distinctive healthcare and education systems [1,3–6,51]. Mistaken intermixing of peer, near-peer, group, mosaic, patient, family, youth, leadership, and novice mentoring which display distinct features, roles and approaches and their conflation with supervision, role modelling, coaching, advising, networking and/or sponsorship in many mentoring studies [52,53] calls into question prevailing understanding of mentoring practice and how it influences mentor training [4,46,54–67]. Concurrently, failure to circumnavigate the restrictions posed by mentorings’ evolving, adaptive, goal-specific, context-sensitive, and mentee-, mentor-, relationship-, and host organization-dependent nature (mentoring’s nature) that limits scrutiny of mentoring programs to those with similar healthcare, educational and clinical settings and congruous mentor and mentee populations has compounded the situation [1,3–6,51].

The lack of an ‘evidence based, user friendly mentor training curriculum’ [23,24,41,43,68], the absence of clear guidelines on the design [69–71] and running of mentor training programs [46,61,72–75], reliance upon data from poorly constructed tools to appraise the training process, and limited support of the mentoring program [46,76–78] also means that most mentor training programs have been carried out on an ad hoc basis creating variability in their quality, intensity, content, and effectiveness [40,43,71,79].

## The need for this review

Growing concerns about lapses in professionalism and abuse of the mentoring process [80–86] have underlined the need to move mentor training from a ‘learned from examples, trial and error and peer observations’ approach to a more organized and regulated process [40,68,87]. A scoping review of mentoring programs in medicine is necessary to explore the potential size and scope of available literature on mentor training in published peer-reviewed and prevailing grey literature [88–92].

With Johnson and Gandhi (2015) commenting that most senior mentors are not trained, the imperative to delineate core training elements and an effective approach to mentor training is evident. Adopting Levac et al.’s (2010) refinement of Arksey and O’Malley’s (2005) framework for scoping reviews, we confine focus upon mentor training within a clearly defined mentoring approach that will better inform subsequent research projects [88,92,93]. Focus upon novice mentoring which is defined as a ‘*dynamic, context-dependent, goal-sensitive, mutually beneficial relationship between an experienced clinician and junior clinicians and/or undergraduates that is focused upon advancing the development of the mentee*’[6] also helps circumnavigate the limitations posed by mentoring’s nature [1,3–6,93,94]. Similarities between novice mentoring practices in undergraduate and postgraduate programs and novice mentoring and research mentoring in medicine which is defined as ‘*a dynamic, collaborative, reciprocal, and sustained relationship focused on an emerging researcher’s acquisition of the values and attitudes, knowledge, skills, and behaviours necessary to develop into a successful independent researcher’* allow them to be considered together [1,3–6,95]. The six steps proposed by Levac et al. (2010) are used to organise our methods and results [90,91,93].

## Stage 1: Identifying the research question

The six-member research team discussed the research question with medical librarians from the medical library at the Yong Loo Lin School of Medicine at the National University of Singapore (NUS), medical librarians at the National Cancer Centre Singapore (NCCS) and local educational experts and clinicians at the NCCS, the Palliative Care Institute Liverpool, Duke-NUS Medical School and Yong Loo Lin School of Medicine. The research question the team decided upon was ‘What is known about mentor training programs in novice mentoring and what are the core elements of the mentor training program?’. All subspecialties of Surgery and Medicine as defined by the Accreditation Council for Graduate Medical Education were included in this study given their common approaches to novice mentoring [96]. Envisioning that the content of the scoping review will guide future research into mentor training, we also considered the comprehensiveness and feasibility of these tools given the longitudinal nature of the mentoring process and the mentor training program. This review adopted a PICOS format to study mentoring programs (Table 1)

**Table 1.** PICOs.

PICOS	Inclusion Criteria	Exclusion Criteria
Population	Medical students/Junior clinicians/Residents	Allied health specialties such as dietetics, nursing, psychology, chiropractic, midwifery, social work
	And Senior clinicians/Attendings/Consultants	
Intervention	Mentor-training program for mentor preparation in the following forms: books, manuals, workshops, lectures and seminars	Non-medical specialties such as Veterinary, Dentistry
	Preparation for new mentors.	
	Refresher courses for current mentors. Mentor-training program or methods in hospital-based or medical school settings or clinical and translational science	Programs using supervision, advisorship, preceptorship, role modelling, coaching, teaching
	Formal or informal mentor-training program/methods.	
	From 1990 – 2018	
Comparison		
Outcome	Outcomes of mentor-training on the mentor and the mentee	
	Reflections and personal experience	
	Program evaluation results from forms and questionnaires	
	Core subjects included in the mentor training program	
	Attitude of mentors and mentees	
	Interprofessional Relations	
	Ethical behaviour	
	Professionalism	
	Publications	
	Grants secured	
	Promotions	
	Problems/barriers of mentoring	
	Mentoring programmes	
	Solutions to current mentor training programmes	
Study design	All study designs and article types were included (observation studies, randomized controlled trials, cohort studies, cross sectional studies, longitudinal studies and case studies)	Non-English language articles

## Stage 2: Identifying relevant studies

Guided by librarians at the NUS' medical library and the NCCS' medical library and by local educational experts and clinicians at the NCCS, the Palliative Care Institute Liverpool, Duke-NUS Medical School, and Yong Loo Lin School of Medicine, the six members of the research team determined the inclusion and exclusion criteria of the review. The broad nature of the research question meant that pilot searches were carried out on variations of the word ‘mentor training’ or ‘frameworks’ that appeared in the title or abstract of articles in medicine. The six members of the research team reviewed the search terms and approved the inclusion/exclusion criteria in the abstract screening tool.

Guided by librarians at the NUS' medical library and the NCCS' medical library and by local educational experts and clinicians at the NCCS, the Palliative Care Institute Liverpool, Duke-NUS Medical School, and Yong Loo Lin School of Medicine, the six members of the research team determined that Pubmed, Embase, ERIC, ScienceDirect, Scopus, Google scholar databases, and a grey literature databases including OpenGrey, Trove, Mednar, Web of Science, Open Dissertations, and British Education Index databases would be used [Refer to Figure 1].

Pilot searches were carried out on variations of the word ‘mentor’ and ‘training’ that appeared in the title or abstract of articles were performed using GreyLit and PubMed databases. Applying the abstract screening tool that the research team designed, the six members of the research team independently read through the abstracts of all the articles identified in the pilot and applied the inclusion/exclusion criteria in the abstract screening tool to the abstracts retrieved. Having discussed the individual results of their pilot searches online and at face-to-face reviewers’ meetings, the six members of the research team reviewed the search terms and employed Sambunjak et al.’s ‘negotiated consensual validation’ approach [97] to achieve consensus on the inclusion/exclusion criteria for the search. The six members of the research team agreed that all research methodologies (quantitative and qualitative) written in English or had English translations would be included. Excluded from the review were articles that did not confine themselves to mentor training in medicine. The search was confined to years 1 January 2000–31 December 2017 as papers before the year 2000 often conflated mentoring with supervision, role modelling, coaching, advising, networking, and/or sponsorship [1,3–6]. There was little change to the inclusion/exclusion criteria following the pilot searches.

The finalised search strategy included the keywords: ‘mentor training’, ‘mentorship training’, ‘mentoring competency’, ‘faculty development’ AND ‘medical faculty’ and their combinations and used in all databases. The same keywords were used for all the databases. All allied health specialities (e.g., dietetics, nursing, psychology, chiropractic, midwifery, social work) and non-medical professions (e.g., science, veterinary, dentistry) were excluded. Mentoring training in peer, near-peer, group, mosaic, patient, family, youth, leadership, mixed and e-mentoring, role modelling, coaching, supervision, networking, and/or advising were excluded.

## Stage 3: Selecting studies to be included in the review

The five members of the team adopted similar search strategies to carry out independent searches for articles about mentoring training in novice mentoring involving the agreed upon databases. All searches were carried out between the 18 July 2018 and the 18 September 2018. Using inclusion and exclusion criteria of papers agreed upon by the research team, titles and abstracts of the papers were independently reviewed by each member of the research team. To ensure concordance and consistency in the search approach, each author examined approximately 50 titles and abstracts using the same search terms, database, and the abstract screening tool and compared their results in online discussions with all the team members. Once clarity on the search terms, inclusion, and exclusion criteria were established, the reviewers proceeded to carry out their independent searches of the agreed upon databases. For the first database being searched, each member of the review team worked on the same batch of 200 titles and abstracts and compared their findings at a weekly face-to-face meeting that were supplemented by online discussions in the event consensus could not be attained and the full-text had to be downloaded and discussed by all the team members. The six-member research team used Sambunjak et al.’s ‘negotiated consensual validation’ approach [97] to achieve consensus on the final list of articles to be included in the scoping review [Refer to Figure 1].

All the articles from the final list of included articles were then downloaded and reviewed independently by each author for inclusion into the study. Each study team member then imported the titles meeting the inclusion criteria into EndNote, to remove duplicates, organise the references, and create their individual lists of abstracts to be studied. The individual lists were discussed at research team meetings where Sambunjak et al.’s (2010) approach of ‘negotiated consensual validation’ [97] was used to reach consensus on a final list of abstracts to be studied.

## Stage 4: Data characterisation and analysis

To carry out a comprehensive review of what is known of mentor training in novice mentoring requires comparing the findings of diverse studies. This process is complicated by mentoring’s nature which limits studies to mentoring programs in similar specialities, settings, healthcare and educational settings, and mentor and mentee populations. Similar obstacles have been faced with studies of mentoring approaches, the matching process that pairs mentees and mentors, Mentoring Relationships (MRs), and the mentoring culture. To circumnavigate these restrictions, studies of matching, MRs and mentoring culture have adopted Braun and Clarke’s approach to thematic analysis [98] to identify consistencies across dissimilar settings, goals and mentee and mentor populations. Braun and Clarke’s approach to thematic analysis [98] also circumnavigates a wide range of research methodologies present amongst the included articles which prevent the use of statistical pooling and analysis [98–100]. Thomas et al. (2014) also highlights that thematic analysis is necessary when studying socio-culturally influenced educational processes such as mentoring.

To evaluate the consistent elements within prevailing mentoring tools and discern the approaches that underpinned the design of these tools the five-members research team worked independently constructing ‘codes’ from the ‘surface’ meaning of the same 15 included articles. Thematic saturation was attained after nine articles. The coding process began with line-by-line coding, followed by focused coding ‘evolving to produce categories that responded to these codes [101]. The authors discussed their analysis online and at the author’s meeting to agree upon a common coding framework and code book using Sambunjak et al.’s ‘negotiated consensual validation’ approach [97]. Using the common coding framework and code book, the authors independently coded the articles from the ‘surface’ meaning of the data ‘across the entire data set’ and the ‘detail rich’ codes were grouped together to identify semantic themes [98,102]. The data was regularly reviewed to ensure all relevant excerpts were coded [98,103]. The themes, coding framework, and code book were also constantly reviewed as part of the iterative process employed within the study [3,98,103]. The authors met face-to-face and online to discuss their themes and Sambunjak et al.’s ‘negotiated consensual validation’ approach [97] was employed to achieve consensus on the final list of themes.

To highlight the wealth of data being reviewed, Arksey and O’Malley’s (2005) descriptive-analytic method was employed [88,93,104] to help contextualize the articles being reviewed and to help the coding process. The descriptive-analytic method records information about the ‘“*process” of each programme or intervention included in the review so that its “outcome” is contextualized and more understandable to readers*’ [88]. To ensure consistency in this process and comparability with prevailing reviews, the authors adapted the data charting form used by Tan et al. (2018) to chart the characteristics of all 49 articles included in this scoping review which included author details, year of publication, purpose of the study/research question, practice setting, methodology, population characteristics, and outcome evaluation (variables of evaluation, evaluation responses, and effectiveness of implementation). (Table A1)

## Stage 5: Collating, summarising, and reporting the results

In total, 4602 titles were reviewed, 3355 abstracts were scrutinised, 232 full-text articles were identified, and 68 articles were included in this scoping review. In keeping with Arksey and O’Malley (2005) and Levac et al.’s (2010) recommendations, this section will consider the ‘numerical and thematic analysis’ [88,93].

## Themes

Thematic analysis revealed four themes: structure, content, outcomes, and evaluation of the mentor training program.

### Structure of mentor-training program

All mentor training programs were designed and supported by a host organization [105]. Abedin et al. (2013) found that of the 55 Academic Health Centres surveyed, 96% provided mentor training at a program level and 93% at an institutional level. The general goals of the programs were to train and support mentors [2,6–11,13,15,17,30,48–51,70,77,81,82,86,95,106,107] and ensure a consistent mentoring experience for mentees [108,109]. McCulloch et al. (2015) and Rhodes (2013) found that mentor training programs also motivate mentors.

Design of mentor-training programs pivots on three domains. The first domain relates to the host organization and its goals, curriculum, mentoring approach, support of the program and its mentoring guidelines, professional standards, and codes of conduct. The second domain relates to the mentor and their availability, motivations, interests, competencies, abilities, experiences and sometimes competing for duties [7,9–11,15,17,30,46,50,95,106]. The third domain considers the trainees and their needs, motivations, goals, abilities, experiences, commitment, and desired characteristics of a mentor [7,9–11,15,17,23,30,50,95,106,110]. Tillman et al. (2013) noted that to meet the mentees’ desire for mentors with experience, qualifications and academic standings, most programs recruited experienced clinicians-scientists [1,3–6].

#### Design framework of the program

Libby et al. (2016) and Sood et al. (2016) suggested that the design of the mentoring program should be determined by individual competencies of the mentor and mentee and the intuitional factors, whilst Keyser et al. (2008) suggested considering mentor selection criteria, mentor incentives, mentor–mentee relationships and research and professional development.

#### Duration and frequency of mentor-training sessions

Mentor-training was carried out in ‘bursts’ of two to nine sessions per month [11,78,109] or ‘stretched’ over six to nine months and/or over different phases of the mentoring program [76,77]. ‘Burst’ programs last about 2 h whilst ‘stretched’ programs tend to be between 6 h to two full days in duration [73,78,105]. Various combinations of ‘burst’ and ‘stretched’ sessions have been proposed although the rationale for these designs was not specified [11,40,41,76]. Fornari et al. (2014) recommended that training begins at the start of the semester to help prepare mentors for their roles and responsibilities.

#### Modes of delivery

##### One-off orientation program

Most programs mandate attendance of orientation programs and most supplemented these programs with either web-based, face-to-face, and/or peer training [109].

##### Lunchtime sessions

Lunchtime sessions focused on sharing personal experiences and ideas in small groups of four to five individuals [77], interactive case-based discussions or seminars [11,77,105,108].

##### Role-plays

Role-plays were used to demonstrate mentoring skills [105] and constructive peer feedback [76].

##### Small group discussion

Consisting of 6–14 participants small group discussions focused on teaching approaches [77], grant writing [11] and fostering mentor relationships, independence and work–life balance [40], challenges to communication, promoting mentoring of women and under-represented groups, reflections [78], and problem-solving [11,105].

##### Didactic presentations or lectures

External experts were often employed to conduct lectures on cultural sensitivity [77] and professional [76,105], educational,,,, and clinical mentoring [76].

##### Seminars

Schmidt et al. (2010) ran six seminars on the required skills and roles of the mentor [107] whilst Feldman et al. (2012) employed 10 case-based seminars over 5 months delivering skills-based exercises, case discussions and ‘key information relevant to all mentors’. The ‘Entering Mentoring' seminar discussed strategies, created a forum for discussions and provided an opportunity for reflection [40,41,43].

##### Workshops

Workshops last a day or two and were more didactic [40] and theoretical [111], addressed practical issues [73] or special interest topics [73,78,105,108,111], updates to the program, interpersonal, communication, organizational, diversity training and knowledge and skills training, and guidance to new mentors [11,12,17,45,110,112].

Johnson and Gandhi (2015) reported improved mentoring competencies, self-efficacy scores and confidence in dealing with diversity following a 2-day workshop.

##### Forum

Open forums allowed mentors to discuss their experiences and review changes to the program [32].

##### Mentoring for mentors

Having new mentors shadow and then mentored by senior mentors provides training and support for new mentors [11,113,114]. Though pairing mentors to mentees was primarily mentee-initiated and based on research and career interests and/or skills [113], Bland et al. (2015) reported that matching resulted in higher research activity while Shollen et al. (2014) found that mentee-initiated matching resulted in increased career satisfaction. A combined matching process has been proposed as a means of reducing mismatches and failed relationships [115,116]. Feldman et al. (2012) reported the use of an online platform to facilitate the creation of mentoring networks that provide mentees with specific, accessible and timely support [117–119].

### Training content

The importance of appropriate training content is underlined by evidence that up to 15% of mentoring relationships failed as a result of mentors not understanding their obligations [115]. Only three [120] mentor training programs were ‘field tested’ [11,40]. The topics covered were determined by the program developers and the ‘general’ [73,74,78,109] and ‘specific’ needs of the mentors [77].

Selection of ‘specific’ mentoring topics was informed by the particular needs, context, and goals of the mentoring program, the mentor’s experience and knowledge on the topic, the characteristics and needs of the mentee and the problems faced by mentees, mentors, and mentoring relationships within the program [11,77,114]. In the clinical research setting, for example, mentors were trained to promote interdisciplinary research, research design, grant writing [77], clinical research skills, and preparation of articles for publication [95,121].

The topics covered in general mentoring skill training include communication skills [122], aligning expectations [11,78,95,109], establishing and overseeing compliance of codes of conduct, standards of practice, timelines and the roles and responsibilities of mentors and mentees [71], assessing professional development [114], and mentoring progress [78,95,109]. Additional topics include promoting professional development, handling difficult scenarios, challenging mentees effectively, nurturing mentoring relationships [12], addressing diversity-related issues [45,123], fostering independence [40,46,73,77,78,97,105,111], role modelling, networking, career advising and guidance on publications, promotions, departmental politics and addressing psychosocial issues [114,119]. Pfund et al. (2014) adapted the Entering Mentoring [124] program that focuses on maintaining effective communication, aligning expectations, assessing understanding and diversity, fostering independence and professional development and articulating a mentoring philosophy and plan [16] to the clinical research setting and found an improvement in competencies in all domains.

General teaching skills training include how to deliver curriculum content, use of educational tools and new approaches [78], demonstration of practical skills through role-play and case discussions [77,78,105], debriefing and providing and receiving feedback [46,77,111]. Other topics also include interpersonal, motivational, coaching, research, self-efficacy, networking, ethics, disciplinary, teaching and technical skills training [95], active listening, building relationships, career guidance, and cultural sensitivity [16,27,95,112].

#### Augmenting the mentor training program

Many programs augment mentor training programs with the peer, near-peer, online [120], ‘functional’ (skills based or project-based mentoring) [29,113], mentoring committees and mentoring networks. Mentor consultation services and mentoring committees [11] provide mentees with the additional mentor and peer mentor support whilst mentoring networks [29] provide a mix of peer and faculty support. Online services [122,125] provide mentors and mentees with supplemental resources including information on best mentoring practices and links to resources, such as mentoring agreements and individual development plans.

#### Peer support network

A support framework for mentors during the training such as the Peer-Onsite-Distance model helps mentors network and support one another [46].

### Impact of mentor-training programs

Mentor training boosts confidence in aligning expectations, working with diverse mentee groups and nurturing mentoring relationships [77,78,105], improved communication skills, providing negative feedback, and addressing ‘difficult conversations’ [12,76–78,105,123]. Tsen et al. (2012) found that mentors reported a renewed sense of enthusiasm after the mentor training program [77].

Long-term benefits [11,30,40,113] include increased effectiveness, improved mentoring skills, academic productivity, increased promotions, awards and grants awarded [108,123].

#### Obstacles to mentor-training programs

The main obstacles to mentor training programs are a shortage of trained mentors, a lack of appreciation of mentor training and a lack of time, funding and accountability in designing and coordinating the mentor-training program [27,30,115,121,122,126]. The role of the host organization in supporting these programs also remain poorly elucidated [27,115,122,127].

### Evaluating mentor-training programs

Investment in time and resources demand evaluation of mentor training programs. Yet many prevailing assessment tools are limited to pre- and post-assessments and self-rated tools [45,74,76]. Anderson, Silet and Fleming’s (2012) apprised mentee empowerment and training, peer learning and mentor training, aligning expectations, mentee program advocate, mentor self-reflection, and mentee evaluation of mentor whilst Johnson et al. (2010) evaluated the sustainability of the program as long-term measures of the success of a mentor training program.

#### Surveys

Anonymized mentor surveys have been used to identify the most and least effective parts of the training program, changes in the participants’ views over the course of the program, and suggested improvements for future training sessions [77,78]. Surveys also assess participants’ satisfaction, the abilities of the facilitator or presenter [105,108], the relevance of the teaching material and extent to which the training objectives were met [76–78].

Surveys 6 months after the program [77,108] or at the latter part of a 6-month-long training program [76] are used to capture changes to the mentor’s life and work [76], mentor competency, and mentoring effectiveness [108].

##### Program assessment tools

Keyser et al.s’ (2008) ‘Self-Assessment Tool for Documenting/Monitoring Institutional Roles in Supporting Research Mentorship’ attempts holistic and longitudinal appraisal of the mentoring program.

The Mentoring Competency Assessment measures changes [12,41,122,125] in communication, aligning expectations, assessing understanding, addressing diversity, and fostering independence [69,125].

The Mentorship Knowledge Test assesses content-specific knowledge [103] whilst Lau et al. (2016) compared baseline and post-workshop mentorship competencies.

## Stage 6: Undertaking consultations with key stakeholders

Stakeholders were consulted on the findings of this scoping review. To highlight the strength of the data to garner and to underline the critical nature of the findings, a quality appraisal of the articles was carried out using the Medical Education Research Study Quality Instrument (MERSQI) and the Consolidated Criteria for Reporting Qualitative Studies (COREQ) (Table A1). Whilst scoping reviews are not traditionally associated with quality assessments, the pool of papers identified in the searches were deemed sufficiently robust to undergo quality assessments. To contextualise the findings, the stakeholders were presented with the table created as part of Arksey and O’Malley’s (2005) descriptive-analytic method [88].

This contextualised and quality appraised data enhanced discussions with stakeholders and garnered their views on the relative importance of a mentor training program, the cost-effectiveness and viability of implementing the findings and also aided input into focus for future studies.

## Discussion

This scoping review highlights the increasing importance placed upon mentor training [95] in novice mentoring [7–11,15]. However, despite evidence that mentor training improves knowledge, skills and attitudes and the need to support mentors in diverse settings [7,9–11,15,17,27,30,50,95,106,121] to meet the changing needs of their mentees [7,9,10,15,17,30,50,106], adoption of mentor training programs has not been uniform particularly in clinical medicine, and informal mentoring programs. This scoping review also sketches the range of available mentor training programs and their content and approaches, the domains being evaluated and the key obstacles to effective mentor training. In so doing, this scoping review highlights the variability in the construct, content and focus of mentor training programs which inevitably impacts practice and oversight of mentoring programs, relationships, and processes [128].

Whilst it was our intention to appreciate the scope of available literature and tools on mentor training in novice mentoring, it is evident that this mere sketch albeit an expansive one lacks depth [128]. This limitation is largely the result of incomplete reporting of prevailing mentor training approaches, the contents of these programs, the manner that the training and the trainees are evaluated and how the program is able to provide effective support for mentors in training. Further limitations to this review lie in this scoping reviews’ adoption of a pragmatic approach that balances practicality and available resources and focuses upon publications in English and those that had English translations. A further limitation to the generalizability of the findings of this scoping review is the presence of a small pool of papers included in this scoping review and the preponderance of American and European papers that limit their global impact. Reliance upon self-rated scales [41] and tools still rooted in ‘*Cartesian reductionism and Newtonian principles of linearity*’ [129] that fail to consider the evolving nature of the mentoring process [1,3–6] also underline the need for a systematic reviews.

However, despite these limitations, this scoping review was carried out with the required rigour and transparency advocated by Arksey and O’Malley (2002), Levac et al. (2009, 2010) and Pham et al. (2014). Use of Endnote a bibliographic manager ensured all the citations from the various databases were properly accounted for.

We believe the findings of this scoping review will be of interest to educators and program designers in undergraduate and postgraduate settings and will help inform the design of future mentor training programs.

The lack of consensus and gaps in evidence on the best approach, necessary content, quality assessment of the programs and evaluation of the competence of mentors following training, also underscore the need for a systematic review of mentor training in medicine.

### Directions for future research

Actualising a consistent mentor training program depends on evaluations of the mentoring process. To facilitate this goal, a critical area for future study must be a design of effective tools to assess mentoring competencies and efficacy longitudinally and holistically. It is only thus, can mentoring in medical education develop to its full potential.

## Appendix

**Table A1.** Summary of Papers with MERSQI and COREQ Scoring.

Study	Study Details	Intervention	MERSQI Score	COREQ Score
Lau et al. (2016)	Qualitative and quantitative Intervention, pre and post survey Single center study at Department of Psychiatry and Behavioural Neurosciences at McMaster University N = 43 mentors	Mentor training workshop for faculty, staff and trainees	14	5
Wahab et al. (2016)	Systematic review	NA	NA	NA
Varma et al. (2016)	Qualitative Case study, post survey Single centre study at Pramukhswami Medical College, India N = 28 mentors, 18 (64.3%) completed feedbacks, 10 (35.7%) partial completion of feedback	Workshop for mentor training	NA	5
Geraci et al. (2017)	Symposium review article for the Medical Education and Educators Symposium	NA	NA	NA
Kashiwagi et al. (2013)	Systematic review of mentoring programs in medical school in USA	NA	NA	NA
Fornari et al. (2014)	Quantitative study; Intervention Multi-centre study N = 14 US Medical schools; 7 schools had preliminary accreditation, 3 had provisional accreditation, 3 were applicant schools, and 1 was a candidate school.	Mentoring programs in the 14 medical schools were evaluated through a survey, comparing the development of these established programs.	13.5	NA
Phitayakorn et al. (2016)	Review article (program evaluation) on mentoring program in Massachusetts General Hospital Department of Surgery	Mentors participated in orientation sessions as part of the mentorship program	NA	NA
Lin et al. (2013)	Quantitative Cross-sectional survey design; Multi-centre study at John Hopkins University, University of Northern Carolina, Medical College of Wisconsin; N = 48 trainees and 61 faculty members	A 20-item survey was designed for otolaryngology residents and faculty of the three centres involved, to evaluate areas for improvement in mentoring in these programs.	10.5	NA
De Dios et al. (2013)	Quantitative Case study; Single centre study in Brown University N=14 participants, 12 (85.7%) completed post workshop survey	A diversity mentoring program was established to increase retention of diverse trainees. Training Workshop for Multicultural Faculty Mentoring was coordinated by the Diversity Committee.	10.5	NA
Tsen et al. (2012)	Quantitative, Single-centre study at Brigham and Women’s Hospital Pre-survey, Intervention, Post-survey N = 16 participants	The Faculty Mentoring Leadership Program (FMLP) is a peer-learning skill development course held monthly from October 2009 to June 2010.	8.5	NA
Kirresh et al. (2011)	Review article on mentoring program	Mentor training sessions	NA	NA
Lewellen-Williams et al. (2006)	Quantitative, Single-centre study at University of Arkansas for Medical Sciences (UAMS) College of Medicine; Intervention; N = 9 potential peer mentors, 22 potential mentees	Design of a questionnaire based on the results of a literature review and structured interviews with administrators from higher education institutes; which was administered to Under-Represented Minority (URM) faculty, housestaff, students.	10.5	NA
Coyne et al. (2006)	Review article on mentoring of new Genitourinary Medicine consultants of the Royal College of Physicians, United Kingdom	A prospective collaboration between Royal College of Physicians (RCP) and Genitourinary medicine consultants to develop a training programme for mentors	NA	NA
Selwa et al. (2003)	Quantitative Case Study Single-centre study at University of Michigan N = 77	Gilman Mentoring Survey	8.5	NA
Connor et al. (2000)	Quantitative Case study Multiple-centre study at Northern and Yorkshire Region Doctors’ Development and Mentoring Network in a regional mentoring scheme N = 83 senior doctors from 4 programmes, 71 (86%) responded to survey	A regional mentoring scheme was formulated to develop mentoring skills, provide a forum for development and establish a mentoring network. A post-program questionnaire evaluated multiple aspects of this program.	11	NA
Freeman et al. (1997)	Case Study Regional initiative in South Thames (West); Intervention, post-survey N = 25 mentors, 68 mentees	The Reflective Cycle model of mentoring, post mentor-training survey	NA	NA
McGee et al. (2016)	Commentary on how race, gender and status diversities impacts mentoring in research fields	NA.	NA.	NA.
Donato et al. (2012)	Opinion Paper on ACGME framework and framework development	Development of a framework for faculty mentoring	NA.	NA.
Feldman et al. (2012)	Mixed-method study to assess long term impacts of mentor training programs in University of California, San Francisco (UCSF) and Translational Science Institute. N = 38 mentors. Survey respondents = 38 (100%)	A survey questionnaire was conducted to assess how the mentor development program they completed impacted their mentoring satisfaction, skills and knowledge.	9	11
Johnson et al. (2014)	Quantitative study to assess the effectiveness of a 2 day mentoring workshop at the University of California, San Francisco (UCSF). N=34 participants. Survey respondents = 34 (100%)	Mentoring the mentor workshop with a pre and post workshop evaluation	13	NA
Chen et al. (2016)	Mixed-method study to assess the efficacy of a paediatric mentoring program at the Stanford University School of Medicine. N = 79 participants. Survey respondents = 89% over 7 years.	A paediatric mentoring program with 7 yearly post-surveys .	10	15
Sorkness et al. (2015)	Narrative paper to describe the structure and activities of the National Research Mentoring Network (NRMN)	Training programs are initiated at a institutional, regional and national level.	NA	NA
Pfund et al. (2013)	Mixed method study to evaluate a new research mentor training curriculum. N = 144 participants. Survey respondents = 135 (94%)	The mentor training curriculum was implemented across 16 different institutions. Data was collected during training sessions via reflective writing and a survey at the end.	11.5	9
Steinert et al. (2009)	Systematic review to synthesise existing evidence on the effects of faculty development interventions.	NA	NA	NA
Singh et al. (2018)	Commentary on student mistreatment in medical education and its long term impacts			
Keyser et al. (2008)	Commentary on how institutions can help support research mentorship via 5 key domains.	NA	NA	NA
Johnson et al. (2010)	Commentary describing a new Mentor Development Program at the University of California, San Francisco on effective mentoring.	The program includes 10 mentoring sessions with different objectives each.	NA	NA
Steinert et al. (2004)	Mixed method study to assess the relationship between mentorship and research productivity. N = 215 participants. Survey respondents = 139 (65%)	Survey to evaluate participants of the National Research Service Award (NRSA) pri-mary care research programs	12.5	13
Shea et al. (2011)	Investigation to determine how career development awardees are selected and how they are subsequently mentored and supported.	NA	NA	NA
Kirsch et al. (2018)	Mixed method study to evaluate a new career mentoring program at University of Minnesota Medical School. N = 30 participants. Survey respondents = 23 (77%)	Career mentoring program with survey evaluations along the course of the program	12.5	8
Harris et al. (2010)	Review article of issues raised by Competency-Based Medical Education (CBME)	NA	NA	NA
Blanchard et al. (2015)	Commentary highlighting the problems and proposed solutions for quality Medical Education Research	NA	NA	NA
Pfund et al. (2016)	Commentary outlining the importance of effective mentoring relationships and frameworks to attain them.	NA	NA	NA
Abedin et al. (2012)	Qualitative research to derive a list of actionable competencies for mentors. This will be used to guide future research directions.	Focus groups, expert panel processes and review of literature, curriculum and mentor evaluation forms were conducted.	NA	10
Gandhi et al. (2014)	Qualitative study to evaluate the effectiveness of a mentoring workshop for HIV Research held at University of California, San Francisco. N = 26 participants Survey respondents = 26 (100%)	2- day workshop at the UCSF to train HIV researchers in US on effective mentoring. A survey was done at the end of each day to evaluate the activities.	11	NA
Ilic et al. (2014)	Quantitative study to analyse the use of a new ACE tool in Evidence Based Medicine (EBM) trainees.	Using an ACE tool developed by the authors to assess medical trainees’ competency in EBM.	13	NA
Schmidt et al. (2010)	Quantitative study for students to assess mentors who have participated in a mentor training sessions.	1-day long mentor trainings for medical educators. Medical students then evaluated mentors to compare between those who attended the training and those who didn’t.	10	NA
Anderson et al (2012)	Commentary on methods of evaluating and giving feedback to mentors to improve mentoring relationships and outcomes	NA	NA	NA
Holmboe et al. (2011)	Commentary on faculty development for assessment and evaluation of evolving Competency Based Medical Education (CBME) principles.	5 essential steps to improve were established.	NA	NA
Welch et al. (2016)	Qualitative study to investigate various mentoring practices in Emergency Medicine Departments in United States.	NA	NA	11
Wong et al. (2017)	Qualitative study to evaluate a new co-learning curriculum amongst faculty in the University of Toronto. N = 56 Participants Survey respondents = 56 (100%)	Co-Learning curriculum for residents of various specialties. 30 semi-structured interviews were done along the way to evaluate the program’s effectiveness.	NA	15
Ehrich et al. (2004)	Review of literature regarding positive and problematic outcomes of mentoring programs in education	NA	NA	NA
Ng et al. (2014)	Review article of factors, practices and characteristics of effective mentoring	NA	NA	NA
Gandhi et al. (2016)	Review article of past workshops conducted and emphasising the various needs for mentoring training programs in HIV research	Multiple 2- day workshops at the UCSF to train HIV researchers in US on effective mentoring. A survey was done before and at the end of each day to evaluate the activities.	NA	NA
Libby et al. (2016)	Quantitative study to evaluate whether a formal mentorship program for scholars resulted in increased grant outcomes at the University of Colorado. N = 150 participants. Respondents = 150 (100%)	Clinical Faculty Scholar’s Program (CFSP) where 25 scholars are matched with faculty members. Compared against control group of 125 members. Outcomes were measured by mean annual counts and dollars of grant awards.	12.5	NA
McCullough et al. (2016	Review article to identify motivations and barriers to improve clinician-educator training programs.	NA	NA	NA
Rhodes, Alexander (2013)	Commentary on benefits, barriers and pathways for essential and successful mentoring in Radiology training.	NA	NA	NA
Santoro et al. (2010)	Quantitative study to evaluate the mentor-protege relationship and its attributes during a training program at the Albert Einstein College of Medicine. N = 188 participants Respondents = 133 (71%)	Clinical Research Training Program (CRTP) that lasts for 2 years where each protege is paired with at least 1 mentor in the same discipline.	9.5	NA
Straus et al (2009)	Qualitative study to characterise relationships between mentor and mentee to facilitate development of future mentorship programs	NA	NA	15
Malina, Debra (2018)	Commentary on Men’s fear of mentoring in light of recent campaigns, and its implications on the future of mentorship in Academic Medicine	NA	NA	NA
DeCastro et al. (2013)	Qualitative study to understand variations in formal mentoring relationships and associated themes.	Interviews were conducted with 100 recipients of Mentored Career Development Awards and 28 of their mentors.	NA	19
Abedin et al. (2013)	Review article to quantify and characterise mentoring practices in awarded Academic Health Centers.	NA	NA	NA
Sood et al. (2016)	Commentary outlining a two-pronged framework for mentor development at an individual and institutional level	NA	NA	NA
Byerley, Julie (2018	Commentary on the challenges of mentoring in light of recent sexual harassment movements	NA	NA	NA
Walters et al. (2017)	Commentary on mentees from diverse backgrounds and the need for mentor training in these minority groups, specifically in HIV research.	NA	NA	NA
Sakushima et al. (2015)	Qualitative study to investigate the quality and availability of mentoring relationships in Japan.	A cross-sectional survey was sent to mentees from 6 Academic Medical Centres in Japan to gain varied responses.	NA	9
Adams, Traci (2016)	Commentary on the positive impacts of mentor training programs in the healthcare industry	NA	NA	NA
Tillman et al. (2013)	Quantitative study on policies and activities that support mentoring activities in US Academic Health Centres. N = 55 participants Respondents = 51 (92%)	NA	12	NA
Hernandez et al. (2017)	Quantitative study analysing a mentoring program for female STEM majors in 7 universities. N = 116	A weekend workshop was initiated for students where they will be able to get mentoring support from faculty members.	15	NA
Shollen et al. (2014)	Quantitative study investigating relationship between mentoring behaviour, satisfaction and productivity for faculty members at the University of Minnesota.	Formal and informal mentoring techniques	10.5	NA
Sorkness et al.	Commentary on a new mentor training curriculum at the University of Wisconsin – Madison.	Curriculum comprising of four 2-hour sessions to train mentors.	NA	NA
Yin et al. (2015)	Commentary on various factors (such as formal mentoring) that facilitate the training of future clinical researchers	NA	NA	NA
Tan et al. (2017)	Review of various mentoring programs to ensure a consistent consolidated approach to mentoring.	NA	NA	NA
Dath et al (2010)	Commentary on faculty development and its importance in preparing mentors for Competency Based Medical Education (CBME)	NA	NA	NA
Magee et al (2013)	Commentary on a new framework to improve the design of Graduate Medical Education surveys so as to achieve quality results.	NA	NA	NA
Pfund et al. (2014)	Quantitative study to determine if a structured mentor curriculum improves research mentoring skills in 16 Academic Health Centres. N = 283	An 8-hour case-based program focusing on 6 core mentoring competencies.	12	NA
Feldman et al.	Mixed method study to evaluate outcomes of a mentor development program at the University of California, San Francisco (UCSF). N = 26 participants	Mentor Development Program consisting of 10 case-based seminars held during monthly half-day meetings over 5 months	10	10
Guerrero et al. (2015)	Review article to design and implement a mentoring and career development initiative to promote diversity in the Biomedical Research field.	NA	NA	NA
